# To degrade or not to degrade: how phase separation modulates selective autophagy

**DOI:** 10.1080/15548627.2025.2476025

**Published:** 2025-03-12

**Authors:** Mariya Licheva, Riccardo Babic, Jeremy Pflaum, Hector Mancilla, Florian Wilfling, Claudine Kraft

**Affiliations:** a Institute of Biochemistry and Molecular Biology, ZBMZ, Faculty of Medicine, University of Freiburg, Freiburg, Baden-Württemberg, Germany; b Faculty of Biology, University of Freiburg, Freiburg, Baden-Württemberg, Germany; c Spemann Graduate School of Biology and Medicine (SGBM), University of Freiburg, Freiburg, Baden-Württemberg, Germany; d Mechanisms of Cellular Quality Control, Max Planck Institute of Biophysics, Frankfurt am Main, Hessen, Germany; e CIBSS - Centre for Integrative Biological Signalling Studies, University of Freiburg, Freiburg, Baden-Württemberg, Germany

**Keywords:** Aggrephagy, Atg11/RB1CC1, autophagy, cargo receptor, initiation hub, phase separation

## Abstract

Selective macroautophagy/autophagy relies on newly formed double-membrane compartments, known as phagophores, to sequester and recycle diverse cellular components, including organelles, biomolecular condensates and protein aggregates, maturing into autophagosomes that fuse with the vacuole/lysosome. Autophagosomes originate at the cargo-vacuole/ER interface, where autophagy factors assemble into the phagophore assembly site (PAS). However, how autophagy proteins organize on the surface of structurally and biophysically different cargoes, and achieve spatial confinement at the PAS to support autophagosome formation remains unclear. Mechanisms governing cargo selection are also poorly understood. In this study, we demonstrate that receptor mobility, driven by low affinity cargo-receptor interactions, is crucial for rendering cellular structures degradable by autophagy. We show that cargo surface mobility, combined with the phase separation of scaffold proteins, drives the formation of early PAS precursors, termed “initiation hubs”. These hubs dynamically rearrange at the cargo-vacuole/ER interface to promote autophagosome biogenesis, providing new insights into selective autophagy initiation.

**Abbreviation:** Ape1: aminopeptidase I; Atg: autophagy related; Cvt pathway: cytoplasm-to-vacuole targeting pathway; GBP-GFP: GFP binding protein-Green Fluorescent Protein; ENDs: Ede1-dependent endocytic protein deposits; ER: endoplasmic reticulum; PAS: phagophore-assembly site; RB1CC1/FIP200: RB1-inducible coiled-coil 1; SQSTM1/p62: sequestosome 1; ULK1: unc-51 like kinase 1.

## Main text

Biomolecular condensates are membrane-less, multi-component assemblies formed through liquid-liquid phase separation, allowing specific biomolecules to compartmentalize without the need for phospholipid membranes. The assembly and clearance of these dynamic structures must be tightly regulated to sustain cellular function, with selective macroautophagy (hereafter autophagy) emerging as a key catabolic pathway for their turnover. Studies in both yeast and mammals have shown that autophagy targets various phase-separated structures, including the Ede1-dependent endocytic protein deposits (ENDs) and aminopeptidase I (Ape1) in yeast, and SQSTM1/p62 in mammals. While the liquid-like properties of these condensates are considered important for their autophagic degradation, some findings suggest that solid protein aggregates can also be cleared through autophagy, implying that cargo liquidity may not always be essential. Overall, autophagy shows remarkable flexibility, efficiently degrading both membrane-less protein assemblies and membrane-delimited organelles including mitochondria, peroxisomes, and portions of the endoplasmic reticulum (ER).

Despite their structural and biophysical differences, all these autophagy cargoes rely on a conserved autophagy machinery for their sequestration within a double-membrane compartment, the autophagosome. However, it remains unclear how autophagy targets such a broad range of cargoes for degradation or what defines a structure as an autophagy cargo. Specifically, it is unknown why some disease-associated protein aggregates and membrane-less condensates are selectively cleared by autophagy while others are not. Furthermore, the molecular and biophysical mechanisms behind the dynamic assembly of the core autophagy machinery during autophagy initiation are poorly understood, particularly how this multi-protein platform is spatially organized at the vacuole (in yeast) or the ER (in mammals) to facilitate autophagosome formation around large and diverse cargoes. We investigated how the biophysical properties of protein condensates make them suitable as autophagy cargoes, highlighting the role of low affinity interactions and phase separation in selective autophagy initiation in yeast and mammals [1]. These findings shed light on how the same conserved protein machinery packages and degrades diverse cellular structures across species.

We investigated how the biophysical nature of condensates influences their ability to serve as autophagy cargoes, by modifying the intrinsic properties of ENDs in yeast and the SQSTM1/p62 condensates in mammalian cells. Stiffening these condensates using the GBP-GFP tethering system increased rigidity and reduced their autophagic turnover, confirming that condensate liquidity supports effective degradation. In both ENDs and SQSTM1/p62 condensates the receptors Ede1 and SQSTM1 phase separate alongside cargo proteins, suggesting that this phase separation facilitates their capture by selective autophagy. This raised the question of whether cargo liquidity or receptor mobility drives autophagic degradation. To distinguish these factors, we analyzed the yeast zymogen precursor Ape1 (prApe1), the main cargo for the cytoplasm-to-vacuole targeting (Cvt) pathway, which differs from ENDs and SQSTM1 condensates as it does not phase separate with its receptor Atg19. Instead, Atg19 forms a protein layer on the surface of prApe1. Using an *in vitro* bead-binding assay, we demonstrated that Atg19 interacts with prApe1 with low affinity. Strengthening this interaction *in vivo* impairs prApe1 delivery to the vacuole, highlighting receptor mobility, rather than cargo liquidity, as the key determinant in selective autophagy turnover.

**Figure 1.**
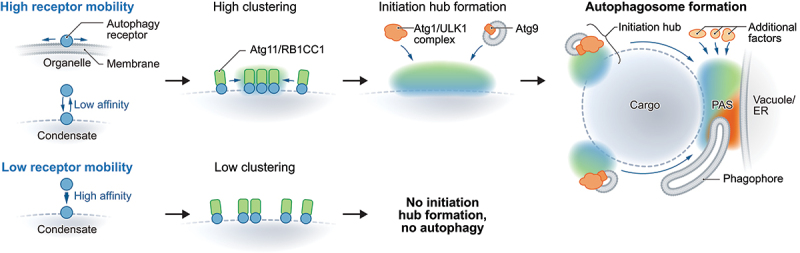
Receptor mobility on cargo surfaces is essential for their uptake by selective autophagy. Membrane-bound cargoes achieve receptor mobility through protein diffusion within their lipid bilayer, while membrane-less cargoes rely on low-affinity interactions between cargo molecules and receptors to create a mobile surface. This mobility supports the phase separation of scaffold proteins like Atg11/RB1CC1 at the cargo surface, driving the formation of initiation hubs. These hubs recruit early autophagy factors, including the Atg1/ULK1 complex and Atg9-containing vesicles, and rearrange at the cargo-vacuole/ER interface upon autophagy induction, where they facilitate productive PAS assembly. The PAS enables phagophore formation around the cargo, ensuring its selective sequestration within an autophagosome. Disrupting receptor mobility impairs initiation hub formation, blocking PAS assembly and, consequently, autophagy.

Cargo engulfment into phagophores begins with the formation of the phagophore-assembly site (PAS in yeast) at the cargo surface. A productive PAS forms in a confined space between the cargo and a membrane compartment, the vacuole in yeast or the ER in mammals, and depends on the scaffold proteins Atg11 (yeast) and RB1CC1/FIP200 (mammals). Atg11 forms low-affinity interactions with the vacuole and core autophagy proteins like Atg9, facilitating PAS assembly through avidity-driven organization. This depends on Atg11’s spatial confinement on the cargo surface. We observed dynamic Atg11 and RB1CC1 foci on various cargoes in yeast and mammals, colocalizing with early autophagy factors such as Atg1 and Atg9 in yeast and ULK1 in mammals. In the Cvt pathway, Atg1- and Atg9-positive Atg11 clusters surround the prApe1 condensate, rearranging toward the vacuole and merging into a single Atg11-positive structure upon autophagy induction. Notably, the late autophagy factor Atg8 localizes exclusively to vacuolar-bound Atg11 clusters before autophagy initiation, suggesting that membrane biogenesis starts at the cargo-vacuole contact site. We termed these early Atg11/RB1CC1 clusters “initiation hubs”, proposing that they function as precursors to a functional PAS. Upon autophagy induction, initiation hubs coalesce at the cargo-vacuole/ER contact site to drive membrane biogenesis (Figure 1).

We tested whether initiation hub formation and reorganization depend on cargo surface mobility. First, receptor mobility on the cargo surface proves essential, enabled by low affinity interactions between receptors and their membrane-less cargoes, or by the lateral protein diffusion within membranes. Second, initiation hub formation is driven by Atg11 phase separation on a dynamic receptor surface. Third, RB1CC1, like Atg11, also links cargo to the autophagosome biogenesis site at the ER. These findings highlight the importance of scaffold proteins, phase separation, and receptor mobility in driving selective PAS assembly during autophagy initiation across diverse cargo types and species.

Our work demonstrates that cargo surface mobility and scaffold protein phase separation drive initiation hub formation, enabling autophagy cargo functionality. Disrupting cargo surface mobility eliminates initiation hubs, blocking autophagy (Figure 1). Applying these principles, we successfully converted a foreign protein particle into a selective autophagy “neo” cargo by introducing low-affinity receptor interactions, restoring surface mobility and initiation hub formation.

In conclusion, these findings can explain why autophagy preferentially targets liquid-like condensates while avoiding certain types of rigid aggregates. We speculate that the phase transition of disease-related condensates into rigid, non-degradable aggregates impairs cargo surface mobility, leading to the loss of initiation hubs and aberrant PAS assembly, ultimately blocking autophagy. This raises the possibility of designing “molecular glues” to render rigid aggregates autophagy-degradable. Understanding how condensate solidification affects protein rearrangement, PAS assembly, and autophagosome biogenesis could provide insights into neurodegenerative disorders.
